# The neonatal mortality risk of vulnerable newborns in rural Bangladesh: A prospective cohort study within the Shonjibon trial

**DOI:** 10.1111/tmi.14092

**Published:** 2025-02-02

**Authors:** Alexandra Hewish, Michael J. Dibley, Shahreen Raihana, Mohammad Masudur Rahman, Sajia Islam, Shams el Arifeen, Tanvir Huda

**Affiliations:** ^1^ Sydney School of Public Health, Sydney Medical School The University of Sydney Camperdown New South Wales Australia; ^2^ Arnold School of Public Health The University of South Carolina Columbia, South Carolina USA; ^3^ Maternal and Child Health Division International Centre for Diarrhoeal Disease Research Dhaka Bangladesh

**Keywords:** birth outcomes, infant, neonatal mortality, newborn, South Asia

## Abstract

**Objectives:**

Preterm birth (<37 weeks), low birth weight (2500 g), small‐for‐gestational‐age (birth weight <10th percentile of a given reference), and large‐for‐gestational‐age (birth weight >90th percentile of a given reference) are indicators of vulnerable infants and risk factors for neonatal mortality. We estimated the prevalence and risk of neonatal mortality associated with these phenotypes and their mutually exclusive phenotypes in rural Bangladesh.

**Methods:**

We conducted a prospective cohort study in five rural districts of Bangladesh using data collected from births in the Shonjibon Trial from 2013 to 2015. We estimated the prevalence of preterm birth, low birth weight, small‐for‐gestational‐age, and large‐for‐gestational‐age infants, individually and for mutually exclusive phenotypes, using a combination of these phenotypes. Neonatal mortality associated with preterm birth, low birth weight, small‐for‐gestational‐age, large‐for‐gestational‐age, and mutually exclusive phenotypes were calculated using Kaplan–Meier survival analysis and Poisson regression for adjusted relative risks (aRR) with 95% confidence intervals (CI).

**Results:**

We included 24,314 live births in this study. The prevalence of preterm birth, low birth weight, small‐for‐gestational‐age, and large‐for‐gestational‐age was 26.2%, 22.9%, 41.7%, and 8.2%, respectively. The prevalence of babies born appropriate for gestational age, with term gestation (≥37 weeks) and normal birth weight (≥2500 g) was 33.3%. For individual phenotypes, the neonatal mortality risk was approximately 3‐fold for preterm, low birth weight, and large‐for‐gestational‐age newborns and 1.5‐fold for small‐for‐gestational‐age newborns compared with appropriate‐for‐gestational‐age, term, and normal birth weight newborns. The risk of neonatal mortality for mutually exclusive phenotypes was highest in small‐for‐gestational‐age, preterm, and low birth weight newborns (aRR = 6.3, 95% CI 4.1–9.6) relative to appropriate for gestational age, term, and normal birth weight newborns.

**Conclusions:**

In rural Bangladesh, most infants are born with one or more vulnerable phenotypes associated with an increased risk of neonatal mortality. Our findings highlight the value of categorising newborns using mutually exclusive vulnerable phenotypes and their neonatal mortality risks, which can be used to tailor interventions to improve survival.

## INTRODUCTION

Globally an estimated 2.4 million infants died during the neonatal period in 2019 [1]. Over the past 30 years, the proportion of under‐five deaths occurring in the neonatal period increased from 40% in 1990 to 47% in 2019 [1]. Around 99% of neonatal deaths occur in low and middle‐income countries [2].

Low birth weight (LBW) (<2500 g), preterm birth (PT) (<37 weeks), and small for gestational age (SGA) (<10th percentile) birth are indicators of newborn vulnerability and risk factors for neonatal mortality [3]. SGA is a proxy for intrauterine growth restriction (IUGR). Annually worldwide, approximately 20 million babies are born with LBW, 15 million preterm, and 23 SGA [4]. South Asia harbours the highest regional prevalence of preterm birth, LBW, and SGA infants [5]. In Bangladesh, approximately 14% are preterm, 31% are SGA, and 28% are LBW [4, 6]. In addition to the increased mortality risk, these infants are at risk of lifelong morbidities, including developmental delays and chronic health problems [3, 7]. There is a lack of data concerning the neonatal mortality risk of large‐for‐gestational‐age (LGA) newborns and their mutually exclusive phenotypes in any setting.

In low‐ and middle‐income countries, there is evidence that small‐for‐gestational‐age (SGA) and preterm newborns have 15 times the neonatal mortality risk compared to appropriate‐for‐gestational‐age (AGA) and term newborns [5]. Vulnerable newborns can be classified in mutually exclusive phenotypes using the parameters of preterm birth, birth weight, and size for gestational age. However, there is a paucity of data on the neonatal mortality risk of these vulnerable newborn phenotypes in Bangladesh and South Asia, which have high prevalences of vulnerable newborns. In 2020, the Lancet launched its Small Vulnerable Newborn Series [8]. Ashorn et al. underlined the value of cateogrising newborns as mutually exclusive phenotypes in this series [9]. These phenotypes differentiate between the contributions of preterm birth, birth weight, and size for gestational age contribute to mortality. It is critical to better understand the mortality risks of vulnerable newborns to effectively target interventions and save lives.

The primary aim of this study was to investigate the prevalence and neonatal mortality risk of preterm, LBW, SGA, and LGA infants separately and for mutually exclusive vulnerable newborn phenotypes. We expected a high prevalence of vulnerable newborns in rural Bangladesh and that neonatal mortality risk would differ between vulnerable newborn types. Furthermore, we hypothesised that newborns with appropriate weight for gestational age, term, and normal birth weight (NBW) would have the lowest neonatal mortality risk. In contrast, SGA, preterm birth, and LBW newborns would have the highest risk.

## METHODS

### Study design

This study was a secondary data analysis of a community‐based cluster randomised controlled trial (Shonjibon Trial) that analysed the effect of iron‐folic acid supplements on neonatal mortality [10]. We examined a prospective cohort of pregnant women up to the neonatal period to describe the prevalence of vulnerable newborns and compare their neonatal mortality risk with those born with appropriate weight for gestational age, term, and NBW in rural Bangladesh. Pregnant women were randomly allocated to the enhanced iron/folic acid distribution programme, receiving daily supplementation with 60 mg elemental iron and 400 μg folic acid starting in the first trimester of pregnancy and sustained for at least 180 days, or were allocated to the usual programme [10]. The usual programme is that pregnant women should receive 60 mg of iron supplementation as recommended by the World Health Organisation and the Government of Bangladesh [10].

### Data source

The Shonjibon Trial was conducted from 2013 to 2015 in five districts of Dhaka (Netrokona, Kishoregan, Mymensingh, Sherpur, and Gazipur), with 31,857 live births. The full trial protocol has been previously published [11]. Trained data collectors who did not implement supplementary intervention gathered the data. Data collectors visited each pregnant woman six times from enrolment to 42 days postpartum. The trial evaluation team collected information using questionnaires on social, economic, demographic, and household characteristics; reproductive history; maternal health; antenatal care; and birth outcomes, including death. The interviewers captured the questionnaire data using special‐purpose programmes on Samsung Galaxy 7‐inch tablets with an internet‐enabled SIM card that transmitted the data to a central database. We constructed a wealth index by using standard demographic and health survey methods.

#### Birth weight

The field team used a portable electronic scale and standard methods to measure birth weight (Weihang, WH‐A08). They measured birth weight in grams within 72 h of delivery. Otherwise, it was extracted from the birth records. We excluded 4138 infants without birth weight from this study.

#### Gestational age

The duration of gestation was measured using the date of the last menstrual period and was confirmed using an on‐spot urine test.

### Definitions

#### Study factors

LBW was defined as birth weight <2500 g, NBW as birth weight ≥2500 g and ≤ 6500g, PT as gestational age < 37 completed weeks, and term (T) as gestational age ≥ 37 weeks. SGA was defined as birth weight < 10th percentile for gestational age, appropriate for gestational age (AGA) between the 10th and 90th percentiles, and large for gestational age (LGA) as >90th percentile of a standard Reference [12]. Newborns were classified into mutually exclusive vulnerable categories based on combinations of birth weight, gestational age, and size for gestational age. These categories are:

**Table:** 

Mutually exclusive categories	Definition
AGA‐T‐NBW	Appropriate‐for‐gestational‐age, term, normal birth weight
AGA‐PT‐NBW	Appropriate‐for‐gestational‐age, preterm, normal birth weight
AGA‐T‐LBW	Appropriate‐for‐gestational‐age, term, low birth weight
AGA‐PT‐LBW	Appropriate‐for‐gestational‐age, preterm, low birth weight
SGA‐T‐NBW	Small‐for‐gestational‐age, term, normal birth weight
SGA‐T‐LBW	Small‐for‐gestational‐age, term, low birth weight
SGA‐PT‐LBW	Small‐for‐gestational‐age, preterm, low birth weight
LGA‐PT‐LBW	Large‐for‐gestational‐age, preterm, low birth weight
LGA‐PT‐NBW	Large‐for‐gestational‐age, preterm, normal birth weight
LGA‐T‐NBW	Large‐for‐gestational‐age, term, normal birth weight

For our analysis, we coded these categories as binary variables: those who were not vulnerable (AGA‐T‐NBW) and those who were vulnerable (remaining categories).

#### Outcomes

Neonatal mortality was defined as death from birth to 28 days postpartum. Stillbirth was defined as birth with no signs of life at or after 28 weeks of gestation, and spontaneous miscarriage was defined as foetal loss before 28 weeks of gestation.

### Statistical analysis

All eligible participants were included irrespective of their intervention status. We excluded birth outcomes with missing birth weight, gestational age, size for gestational age, or groundwater iron. We then examined the distributions of maternal sociodemographic, household, and newborn characteristics potentially associated with neonatal mortality based on the Mosely and Chen child survival framework [13]. The factors we adjusted for were maternal age at delivery, maternal education, husband's education, parity, maternal height, household size, household wealth, mode of infant delivery, immediate and antenatal care, number of foetuses, and parity. Other factors were the working status of the household head, years of schooling of the household head, last birth outcome, mother's middle‐upper arm circumference, and sex of the infant. We used Kaplan–Meier survival analysis to compare survival probabilities to 28 days of life and estimate mortality rates (deaths per 1000 live births) and 95% confidence intervals. We used a multilevel mixed‐effect generalised linear model with a modified Poisson regression [14] with a log‐link function, exchangeable correlation, and robust variance to estimate the adjusted relative risks (aRR) and 95% confidence intervals. The models examined neonatal mortality in vulnerable baby categories, with community cluster and sub‐district strata as random effects, and non‐vulnerable infants (those born AGA‐T‐NBW as the reference group).

We adjusted for the effects of supplementation intervention, cluster randomisation, type of water source, and groundwater iron. We used backward elimination to adjust for additional socioeconomic, demographic, and household characteristics for each vulnerable baby category based on the conceptual framework by Mosely and Chen [13]. Data analyses used Stata version 14.2.

### Ethical considerations

The International Centre for Diarrhoeal Disease Research, Bangladesh (icddr,b) and the University of Sydney ethics committees approved the Shonjibon trial. National technical advisory and data safety monitoring boards were established to safeguard participants' interests and maintain research quality.

## RESULTS

**FIGURE 1:**
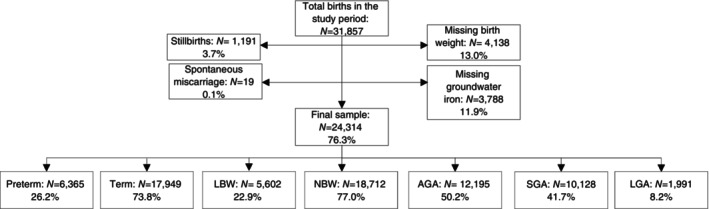
Flow diagram of the study populations. AGA, appropriate‐for‐gestational‐age (birth weight ≥10th percentile and ≤90th percentile of a standard US reference) [12]; LBW, low birth weight (< 2500 g); LGA, large‐for‐gestational‐age (birth weight >90th percentile of a standard US reference) [12]; NBW, normal birth weight (≥ 2500 g); SGA, small‐for‐gestational‐age (birth weight <10th percentile of a standard US reference) [12]; preterm (<37 weeks); term (≥ 37 weeks). The total missing data does not equal the difference between total births and the final sample, as some births were missing multiple variables.

There were 31,857 births during the study period, of which we excluded 4138 due to missing birth weight and 3788 because of missing groundwater iron measurements. There were 1191 stillbirths and 19 spontaneous miscarriages. After excluding 7543 births due to missing data for one or more of these factors, 24,314 live births were included in the analysis (Figure 1).

The prevalences of preterm birth, LBW, SGA, and LGA were 26.2%, 22.9%, 41.7%, and 8.2%, respectively (Figure 1). The highest prevalence of vulnerable newborns was SGA‐T‐NBW (24.5% of all live births) and the lowest was AGA‐T‐LBW (0.1% of all live births). One‐third of all live births were non‐vulnerable newborns (AGA‐T‐NBW).

The social, household, maternal, and birth characteristics were well‐balanced between non‐vulnerable newborns (those born with AGA‐T‐NBW) and vulnerable newborns (all remaining mutually exclusive phenotypes). However, vulnerable newborns had a lower wealth index score, a lower proportion of skilled birth attendants at birth, and shorter maternal height than non‐vulnerable newborns (Table 1).

**TABLE 1:** Characteristics of 24,314 live births included in this analysis by vulnerability.

	Not vulnerable^a^		Vulnerable^b^		Total	
Characteristics	*N*	(%)	*N*	(%)	*N*	(%)
Prevalence	8101	33.3	16,213	66.7	24,314	100.0
Maternal characteristics						
Age at delivery (years)						
12–14	16	(0.2)	43	(0.3)	59	(0.2)
15–19	1535	(19.0)	3553	(21.9)	5088	(20.9)
20–34	6232	(76.9)	11,922	(73.5)	18,154	(74.7)
35+	318	(3.9)	695	(4.3)	1013	(4.2)
Mean age (SD)	24.0	(4.90)	23.8	(5.08)	23.9	(5.02)
Maternal education						
No education	1596	(19.7)	4010	(24.7)	5606	(23.1)
Primary incomplete	723	(8.9)	1715	(10.6)	2438	(10.0)
Primary complete	1465	(18.1)	3407	(21.0)	4872	(20.0)
Secondary incomplete	2971	(36.7)	5405	(33.3)	8376	(34.5)
Secondary complete or higher	1346	(16.6)	1676	(10.3)	3022	(12.4)
Mean years of education (SD)	5.9	(3.77)	5.1	(3.65)	5.4	(3.71)
Husband's education						
No education	2759	(34.1)	6788	(41.9)	9547	(39.3)
Primary incomplete	700	(8.6)	1560	(9.6)	2260	(9.3)
Primary complete	1350	(16.7)	3010	(18.6)	4360	(17.9)
Secondary incomplete	1859	(23.0)	3012	(18.6)	4871	(20.0)
Secondary complete or higher	1430	(17.7)	1834	(11.3)	3264	(13.4)
Mean years of education (SD)	4.9	(4.06)	4.1	(3.75)	4.3	(3.88)
Parity						
1–2	5493	(67.8)	10,680	(65.9)	16,173	(66.5)
3+	2608	(32.2)	5533	(34.1)	8141	(33.5)
Maternal height						
<145 cm	1183	(14.6)	3268	(20.1)	4451	(18.3)
≥145 cm	6918	(85.4)	12,945	(79.8)	19,863	(81.7)
Mean (SD)	150.9	(5.84)	149.9	(6.08)	150.2	(6.02)
Mean household size (SD)	4.5	(2.16)	4.4	(2.02)	4.4	(2.07)
Mean household wealth score (SD)	0.4	(2.75)	−0.2	(2.17)	0.0	(2.40)
Households with no drinking water iron	1849	(22.8)	3476	(21.4)	5325	(21.9)
Households with any drinking water iron	6252	(77.1)	12,737	(78.6)	18,989	(78.1)
Infant characteristics						
Sex of newborn						
Male	4061	(50.1)	8706	(53.7)	12,767	(52.5)
Female	4040	(49.9)	7507	(46.3)	11,547	(47.5)
Mean enrollment gestational age in months (SD)	9.1	(0.40)	8.7	(0.87)	8.8	(0.77)
Pregnancy care						
Mode of delivery						
Vaginal	6013	(74.2)	13,651	(84.2)	19,664	(80.9)
Caesarian	2088	(25.8)	2562	(15.8)	4650	(19.1)
Immediate care						
Skilled Birth Attendant	3549	(43.8)	5237	(32.3)	8786	(36.1)
Unskilled Birth Attendant	4552	(56.2)	10,976	(67.7)	15,528	(63.9)
Antenatal care						
No	1452	(17.9)	3218	(19.9)	4670	(19.2)
Yes	6649	(82.1)	12,995	(80.2)	19,644	(80.8)
Birth outcomes						
Number of live births	8101	(33.3)	16,213	(66.7)	24,314	(100.0)
Multiple foetuses						
Single	8082	(99.8)	15,869	(97.9)	23,951	(98.5)
Multiple	19	(0.2)	344	(2.1)	363	(1.5)
Neonatal death						
<7 days	75	(0.9)	284	(1.8)	359	(1.5)
<28 days	82	(1.0)	339	(2.1)	421	(1.7)

Abbreviations: AGA‐T‐LBW, appropriate‐for‐gestational‐age, term and low birth weight; AGA‐PT‐LBW, appropriate‐for‐gestational‐age, preterm and low birthweight; LGA‐PT‐LBW, large‐for‐gestational‐age, preterm and low birth weight; LGA‐PT‐NBW, large‐for‐gestational‐age, preterm and normal birth weight; LGA‐T‐NBW, large‐for‐gestational‐age, term and normal birth weight. SGA‐T‐NBW, small‐for‐gestational‐age, term and normal birth weight; SGA‐T‐LBW, small‐for‐gestational‐age, term and low birth weight; SGA‐PT‐LBW, small‐for‐gestational‐age, preterm and low birth weight.

^a^ Not vulnerable = AGA‐T‐NBW (appropriate‐for‐gestational‐age, term and normal birth weight).

^b^ Vulnerable = all other mutually exclusive phenotypes combined (AGA‐PT‐NBW = appropriate‐for‐gestational‐age, preterm and normal birth weight).

Most newborns with LGA had the LGA‐PT‐NBW phenotype (Table 2).

**TABLE 2:** Prevalence of mutually exclusive vulnerable newborn phenotypes in rural Bangladesh.

			(95% CI)	
Mutually exclusive phenotype	*N*	% total live births	LBL	UBL
AGA‐T‐NBW	8101	33.3	(32.2, 34.4)	
AGA‐PT‐NBW	2819	11.6	(10.9, 12.3)	
AGA T‐LBW	16	0.1	(0.0, 0.1)	
AGA‐PT‐LBW	1259	5.2	(4.7, 5.7)	
SGA‐T‐NBW	5953	24.5	(23.5, 25.5)	
SGA‐T‐LBW	3409	14.0	(12.9, 15.3)	
SGA‐PT‐LBW	766	3.2	(2.9, 3.5)	
LGA‐PT‐LBW	152	0.6	(0.5, 0.8)	
LGA‐PT‐NBW	1369	5.6	(4.9, 6.4)	
LGA‐T‐NBW	470	1.9	(1.7, 2.3)	

Abbreviations: AGA, appropriate‐for‐gestatational‐age (birth weight ≥10th centile and ≤90th centile of a standard US reference) [12]; AGA‐T‐NBW, appropriate‐for‐gestatational‐age, term and normal birth weight; AGA‐PT‐NBW, appropriate‐for‐gestatational‐age, preterm and normal birth weight; AGA‐T‐LBW, appropriate‐for‐gestatational‐age, term and low birth weight; AGA‐PT‐LBW, appropriate‐for‐gestatational‐age, preterm and low birthweight; LBW, low birth weight (<2500 g); LGA, large‐for‐gestational‐age (birth weight >90th centile of a standard US reference) [12]; LGA‐PT‐LBW, large‐for‐gestational‐age, preterm and low birth weight; LGA‐PT‐NBW, large‐for‐gestational‐age, preterm and normal birth weight; LGA‐T‐NBW, large‐for‐gestational‐age, term and normal birth weight; NBW, normal birth weight (≥2500 g); preterm (<37 weeks); term (≥37 weeks); SGA, small‐for‐gestational‐age age (birth weight <10th centile of a standard US reference) [12]; SGA‐T‐NBW, small‐for‐gestational‐age, term and normal birth weight; SGA‐T‐LBW, small‐for‐gestational‐age, term and low birth weight; SGA‐PT‐LBW, small‐for‐gestational‐age, preterm and low birth weight.

The neonatal mortality rate was the highest for LBW newborns (32.7 deaths per 1000 live births), followed by preterm (32.4 per 1000 live births) and LGA newborns (29.1/1000 live births). SGA newborns had a significantly lower neonatal mortality rate (16.9/1000 live births). SGA‐PT‐LBW newborns had the highest neonatal mortality rate (73.1 per 1000 live births), whereas those born with AGA‐T‐NBW had the lowest at (10.1 per 1000 live births). However, the number of deaths reflected the mortality risk pattern. There were 776 SGA‐PT‐LBW newborns, which accounted for 13.3% of the total deaths, compared to AGA‐PT‐LBW newborns, which had 1259 live births and represented 17.4% of the total deaths (Table 3).

**TABLE 3:** Neonatal mortality rate of vulnerable newborns in rural Bangladesh.

	Live births	Deaths	% of total deaths	Neonatal mortality rate (per 1000 live births)	(95% CI)		
					LBL	UBL	
Inclusive phenotypes							
Low birth weight (<2500 g)	5602	183	43.6	32.7	(28.3, 37.7)		
Preterm (<37 weeks)	6365	206	49.0	32.4	(28.3, 37.0)		
Small‐for‐gestational‐age (<10th centile)^a^	10,128	171	40.7	16.9	(14.6, 19.6)		
Large‐for gestational age (>90th centile)^b^	1991	58	13.8	29.1	(22.6, 37.5)		
Mutually exclusive phenotypes							
AGA‐T‐NBW	8101	82	19.5	10.1	(8.2, 12.6)		
AGA‐PT‐NBW	2819	36	8.6	12.8	(9.2, 17.7)		
AGA T‐LBW	16	0	0.0	0.0	(0.0, 0.0)		
AGA‐PT‐LBW	1259	73	17.4	58.0	(46.4, 72.4)		
SGA‐T‐NBW	5953	66	15.7	11.1	(8.7, 14.1)		
SGA‐T‐LBW	3409	49	11.7	14.4	(10.9, 19.0)		
SGA‐PT‐LBW	766	56	13.3	73.1	(56.7, 93.9)		
LGA‐PT‐LBW	152	5	1.2	32.9	(13.8, 77.2)		
LGA‐PT‐NBW	1369	36	8.6	26.3	(19.0, 36.3)		
LGA‐T‐NBW	470	17	4.0	36.2	(22.6, 57.5)		

^a^ Birth weight <10th centile of a standard US reference [12].

^b^ Birth weight >90th centile of a standard US reference [12].

Abbreviations: AGA, appropriate‐for‐gestational‐age (birth weight ≥10th centile and ≤90th centile of a standard US reference) [12]; AGA‐T‐NBW, appropriate‐for‐gestational‐age, term and normal birth weight; AGA‐PT‐NBW, appropriate‐for‐gestational‐age, preterm and normal birth weight; AGA‐T‐LBW, appropriate‐for‐gestational‐age, term and low birth weight; AGA‐PT‐LBW, appropriate‐for‐gestational‐age, preterm and low birthweight; LBW, low birth weight (<2500 g); LGA, large‐for‐gestational‐age (birth weight >90th centile of a standard US reference) [12]; LGA‐PT‐LBW, large‐for‐gestational‐age, preterm and low birth weight; LGA‐PT‐NBW, large‐for‐gestational‐age, preterm and normal birth weight; LGA‐T‐NBW, large‐for‐gestational‐age, term and normal birth weight; NBW, normal birth weight (≥2500 g); preterm (<37 weeks); term (≥ 37 weeks); SGA‐T‐NBW, small‐for‐gestational‐age, term and normal birth weight; SGA, small‐for‐gestational‐age age (birth weight <10th centile of a standard US reference) [12]; SGA‐T‐LBW, small‐for‐gestational‐age, term and low birth weight; SGA‐PT‐LBW, small‐for‐gestational‐age, preterm and low birth weight.

**FIGURE 2:**
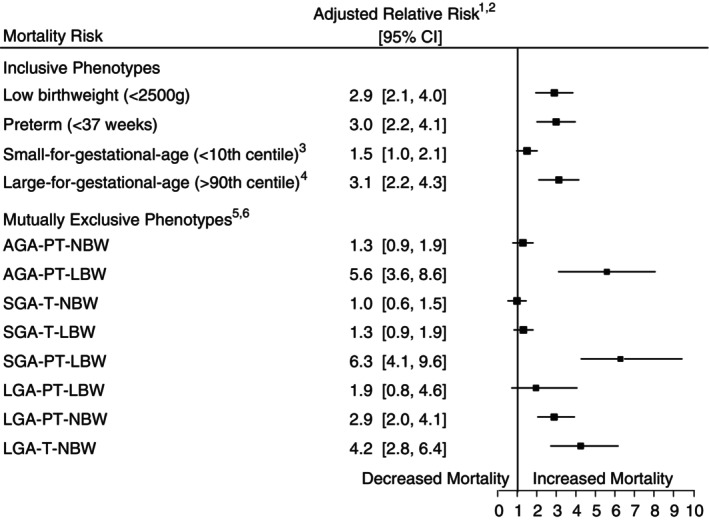
Neonatal mortality risk of vulnerable newborn phenotypes in rural Bangladesh. ^1^Adjusted for effects of the intervention, groundwater iron, and socioeconomics status, maternal and infant characteristics. ^2^Reference group AGA‐T‐NBW. ^3^Birth weight < 10th centile of a standard US reference [12]. ^4^Birth weight >90th centile of a standard US reference [12]. ^5^AGA‐T‐LBW excluded due to no deaths. ^6^AGA, appropriate‐for‐gestational‐age (birth weight ≥10th centile and ≤90th centile of a standard US reference) [12]; AGA‐T‐NBW, appropriate‐for‐gestational‐age, term and normal birth weight; AGA‐PT‐NBW, appropriate‐for‐gestational‐age, preterm and normal birth weight; AGA‐T‐LBW, appropriate‐for‐gestational‐age, term and low birth weight; AGA‐PT‐LBW, appropriate‐for‐gestational‐age, preterm and low birthweight; LBW, low birth weight (<2500 g); LGA, large‐for‐gestational‐age (birth weight >90th centile of a standard US reference) [12]; LGA‐PT‐LBW, large‐for‐gestational‐age, preterm and low birth weight; LGA‐PT‐NBW, large‐for‐gestational‐age, preterm and normal birth weight; LGA‐T‐NBW, large‐for‐gestational‐age, term and normal birth weight; NBW, normal birth weight (≥2500 g); preterm (<37 weeks); term (≥ 37 weeks); SGA, small‐for‐gestational‐age age (birth weight <10th centile of a standard US reference) [12]; SGA‐T‐NBW, small‐for‐gestational‐age, term and normal birth weight; SGA‐T‐LBW, small‐for‐gestational‐age, term and low birth weight; SGA‐PT‐LBW, small‐for‐gestational‐age, preterm and low birth weight.

The mortality risk differed among the mutually exclusive vulnerable baby categories compared to the reference group (AGA‐T‐NBW). The relative mortality risks for preterm, LBW, and LGA newborns were 3.0, 2.9, and 3.1, respectively, compared with those born with AGA‐T‐NBW. Preterm and LBW newborns with SGA or AGA parameters had the highest mortality risks of all phenotypes with aRR of 6.3 (95% CI 4.1–9.6) and 5.6 (95% CI 3.6–8.6), respectively, compared to AGA‐T‐NBW newborns. However, LGA newborns born preterm and LBW only had a 1.9 (95% CI 0.8–4.6) times the mortality risk compared to the reference group. The highest neonatal mortality risk of LGA infants was in those born LGA‐T‐NBW (aRR = 4.2, 95% CI 2.8–6.4) (Figure 2).

## DISCUSSION

In this prospective cohort study, we found that a high proportion of infants born in rural Bangladesh was considered vulnerable, accounting for 66.7% of all births. Specifically, 40% of these infants were classified as SGA, 26% were preterm, and 23% had LBW. Despite making up only approximately 8% of the vulnerable births, LGA newborns had a similar neonatal mortality risk to preterm and LBW newborns. Furthermore, LGA, preterm, and LBW newborns had an approximately three‐fold higher mortality risk than newborns with an appropriate gestational age and normal birth weight (AGA‐T‐NBW), which was higher than the observed 1.5‐fold increased risk in SGA newborns. However, those born SGA‐PT‐LBW had the highest risk of neonatal mortality among all vulnerable newborns, with a more than 6‐fold increased risk of neonatal death compared to AGA‐T‐NBW newborns.

### Significance of this study

No other published articles have detailed mortality risks associated with mutually exclusive categories of vulnerable infants in rural Bangladesh or South Asia, as outlined by Ashorn et al. outlined [9]. The difference in mortality risk between these categories provides a more detailed picture of the interplay between preterm birth, LBW, and SGA, which are well‐known risk factors for neonatal mortality. In addition, there are no previous descriptions of neonatal mortality risk associated with LGA newborns and their mutually exclusive phenotypes. This study highlights the double burden of vulnerability and shows that both large and small newborns are susceptible to increased mortality. Undoubtedly, these results are of public health significance, as both the prevalence and neonatal mortality risk associated with mutually exclusive phenotypes must be considered when tailoring programmes to reduce neonatal deaths.

### Strengths and limitations

A strength of our study design was the adjustment for important confounders based on the Mosely and Chen conceptual framework for neonatal mortality [13]. Adjusting for maternal sociodemographic, household, and newborn characteristics reduces potential bias in our estimates of neonatal mortality risk. Another strength was that the newborn cohorts were of sufficient size to provide prevalence estimates for all vulnerable and non‐vulnerable phenotypes.

The first limitation of our study was the sample size, which was not large enough to estimate the mortality for all vulnerable baby phenotypes. We excluded 4138 births due to missing birth weights, representing 13% of the total births.

A second limitation is the possibility of residual confounding, as we had to restrict our analysis to data collected on confounders in the primary Shonjibon trial. Ideally, we should have controlled for other factors such as maternal morbidities or congenital abnormalities. Furthermore, while we appropriately excluded live births with missing data and whose phenotypes could not be determined, we did not analyse the distribution of confounders in the excluded population, which may be a source of selection bias.

A final limitation is that we used a standard US reference to assess newborn size instead of the INTERGROWH‐21st Project guide, a new international standard to describe newborn growth in developed and developing countries [15]. INTERGROWTH has a lower 10th percentile birth weight cut‐off than the common US reference, reduces the number of infants classified as SGA, and increases the proportion of those AGA [4]. Therefore, we may have overestimated the proportion of these infants in our study. The prevalence of SGA in our study (41.7%) was higher than that in 2012, which found that 30.5% of babies were born SGA in Bangladesh [4]. It is also possible that the true mortality risk for SGA newborns is higher or lower than our findings. Despite these limitations, our results are valid.

### Comparison of results with current evidence

Previous reports have established that preterm, SGA, and LBW newborns in low‐ and middle‐income countries have a higher neonatal mortality risk [4, 5, 16]. A pooled country analysis of LMICs found that neonates born preterm in Asia had an aRR of 3.4 (95% CI 2.8–4.0) compared to infants born AGA‐T [5]. This risk was slightly higher than that in preterm infants compared to AGA‐T‐NBW, as in our study, possibly due to the higher neonatal mortality rate in our reference population. Although our aRR for SGA infants includes the null value (aRR 1.5 95% CI 1.0–2.1), the pooled analysis estimated the RR for SGA infants in Asia as 1.6 (95%CI 1.2–2.2) was comparable to our result [5]. Overall pooled RR for LMICs for preterm and SGA were higher than our Bangladesh results and the regional estimates at 6.82 and 1.83, respectively, likely due to the influence of high neonatal death in Latin America [5].

Previous studies have also examined the neonatal mortality risk of mutually exclusive preterm and size‐for‐gestational‐age phenotypes in Asia. They found that infants born SGA and term had the lowest neonatal mortality risk (RR 3.4, 95% CI 2.4–5.0) of the vulnerable newborns [5]. While we observed the same trend in our analysis; SGA‐T‐NBW and SGA‐T‐LBW had the lowest risk of neonatal death, and our results were not statistically significant.

There is little evidence of neonatal mortality risk associated with the phenotypes described by Ashorn et al. in low‐income or middle‐income settings. However, a recent population‐based cohort study in Brazil showed that while the proportion of babies born SGA‐PT‐LBW was small (1.0%), these newborns had the greatest risk of neonatal mortality (HR = 62.0; 95% CI 60.8–63.2) compared to AGA, term and normal birthweight newborns [17]. Analysis in South Asia further supports these results showing that preterm and SGA newborns had the highest risk of neonatal mortality (RR 16.7 95% CI 13.0–21.5) compared with term and AGA nerwborns [5]. These results are consistent with our findings, although rural Bangladesh has a higher prevalence of SGA‐PT‐LBW newborns (3.2%) than in the Brazilian cohort study, which is potentially due to the poorer socioeconomic and nutritional status of Bangladeshi women [18].

The Brazilian analysis also found that babies born with preterm and LBW phenotypes had the highest neonatal mortality rates compared to AGA‐T‐NBW babies [17]. However, their analysis did not include LGA newborns [17]. Our findings showed that while this was true for SGA‐PT‐LBW and AGA‐PT‐LBW newborns, infants born LGA‐T‐NBW had a higher mortality rate than LGA‐PT‐NBW infants. This finding may be due to the lack of data on LGA‐PT‐LBW infants. However, a retrospective observational cohort study in Japan found that LGA babies born extremely premature (<26 weeks) had no elevated mortality risk compared to AGA [19]. There is a need for more data concerning LGA phenotypes and their mortality risks in other comparable settings.

Our analysis showed that LGA newborns had approximately double the risk of neonatal mortality compared with SGA newborns. Although current literature makes no direct comparisons of neonatal mortality between SGA and LGA newborns, in middle‐ and high‐income countries LGA newborns are known to have elevated mortality risks due to birth complications like asphyxia [20]. Research from rural Bangladeshi has shown higher early neonatal mortality from birth asphyxia due to inadequate skilled birth attendance and newborn care [21]. In our study 67.7% of vulnerable newborns had unskilled birth attendance which may explain the increased mortality risk in LGA newborns.

Our results suggest that further research is needed to design methods for predicting or identifying vulnerable newborn phenotypes to improve care during pregnancy. An investigation should also determine the potential causal mechanisms of neonatal mortality by vulnerable newborn phenotypes to optimise nutrition and health outcomes for mothers and babies. Additionally, large knowledge gaps remain regarding neonatal death in LGA infants in low‐ and middle‐income countries, which must be addressed. The variation in neonatal mortality risk across different vulnerable newborn categories suggests that public health programmes in low‐ and middle‐income settings must be versatile and designed for large and small vulnerable phenotypes to reduce mortality and improve child survival.

## CONCLUSIONS

Approximately two‐thirds of babies born in rural Bangladesh are considered vulnerable because they exhibit one or more of the following phenotypes: preterm birth, LBW, small‐for‐gestational age, or large‐for‐gestational age. This vulnerability places them at a significantly higher risk of neonatal mortality, up to three times greater than that in nonvulnerable newborns.

Babies born small‐for‐gestational‐age, preterm, and LBW had the highest risk of neonatal mortality, while those born appropriate‐for‐gestational‐age, term, and NBW had the lowest risk.

This study provides critical information on the classification of births into mutually exclusive vulnerable newborn phenotypes and their associated neonatal mortality risks. These findings may assist in understanding the risk factors for neonatal mortality, which is important for the design and implementation of preventive public health interventions to reduce neonatal mortality and improve child health outcomes across all vulnerable baby phenotypes.

## FUNDING INFORMATION

Funding for this study was provided by the National Health and Medical Research Council (NHMRC). The NHMRC had no role in study design, data collection, analysis, interpretation, or manuscript preparation.
